# Novel organoid construction strategy for non-involuting congenital hemangioma for drug validation

**DOI:** 10.1186/s13036-023-00348-6

**Published:** 2023-04-27

**Authors:** Haoche Wei, Yanan Li, Li Li, Qian Hu, Mingsong Shi, Linbo Cheng, Xile Jiang, Yanting Zhou, Siyuan Chen, Yi Ji, Lijuan Chen

**Affiliations:** 1 State Key Laboratory of Biotherapy and Cancer Center, National Clinical Research Center for Geriatrics, West China Hospital of Sichuan University, Chengdu, 610041 China; 2 Division of Oncology, Department of Pediatric Surgery, West China Hospital of Sichuan University, Chengdu, 610041 China; 3 Med-X Center for Informatics, Sichuan University, Chengdu, 610041 China; 4 Institute of Clinical Pathology West China Hospital, Sichuan University, Chengdu, 610041 Sichuan Province China; 5 Department of Hematology, West China Hospital, Sichuan University, Sichuan, 610041 China; 6 Chengdu Women’s and Children’s Central Hospital, School of Medicine, University of Electronic Science and Technology of China, Chengdu, China; 7 Clinical Nutrition Department, West China Hospital of Sichuan University, Chengdu, 610041 China; 8 Key Laboratory of Basic Pharmacology of Ministry of Education and Joint International Research Laboratory of Ethnocentric of Ministry of Education, Zunyi Medical University, Zunyi, Guizhou, 563006 China; 9 Pediatric Intensive Care Unit, Department of Critical Care Medicine, West China Hospital of Sichuan University, Chengdu, 610041 China

**Keywords:** Non-involuting congenital hemangiomas, Vascular tumor, Patient-derived organoids, Drug validation

## Abstract

**Background:**

Non-involuting congenital hemangiomas (NICHs) are fully formed vascular tumors at birth with distinctive clinical, radiologic, and histopathological profiles. In the literature, there is no effective therapy strategy for patients with NICH except surgery. Currently, no cell line or animal model exists for studying the mechanism of NICH and drug validation. We plan to construct a new strategy by constructing NICH organoids for further study.

**Result:**

Here, we report a novel NICH organoid system construction and optimization process. Both HE and immunohistological staining exactly matched NICH tissue. We further performed transcriptome analysis to elucidate the characteristics of NICH organoids. Both NICH tissue and NICH organoids manifested similar trends in download sites. NICH organoids display novel features to new cells derived from organoids and show spectacular multiplication capacity. In the preliminary verification, we found that cells splitting from NICH organoids were human endothelial cells. Drug validation demonstrated that trametinib, sirolimus, and propranolol showed no inhibitory effects on NICH organoids.

**Conclusion:**

Our data show that this new NICH-derived organoid faithfully captured the features of this rare vascular tumor. Our study will boost further research on the mechanism of NICH and drug filtering in the future.

**Supplementary Information:**

The online version contains supplementary material available at 10.1186/s13036-023-00348-6.

## Introduction

Congenital hemangiomas (CHs) are an uncommon type of vascular tumor that originate in utero and are fully developed at birth, as initially described by Boon et al. in 1996 [1]. CH varies from typical infantile hemangioma (IH), which has a specific proliferating and involuting phase and immunoreactivity for the cell surface marker glucose transporter-1 (GLUT-1) [1]. CHs can be divided into three distinct subgroups: rapidly involuting congenital hemangioma (RICH), non-involuting congenital hemangioma (NICH), and partially involuting congenital hemangioma (PICH) [2, 3]. Generally, it has been reported that the overall prevalence of CH is 17.0 per 10,000 in all newborns [4]. However, the actual prevalence of RICH and NICH is not clear.

RICH is reversible after birth and, in most cases, completely disappears between the ages of 6 and 14 months [5]. NICHs do not regress on their own but rather continue to exist and expand in proportion to the child [2]. PICHs are similar to RICHs in the early stage, but subsequently, they cease regressing, and the residual lesions are unrecognizable from NICHs [6]. Interestingly, a slight enlargement of the NICH over the years has recently been reported. [3] CHs can result in a variety of severe complications, including permanent disfigurement, ulceration, bleeding, obstruction of a vital organ, and congestive heart failure [5].

The pathogenesis of CHs is still unknown. There is no proven safe and effective medical treatment for NICHs; hence, surgery is almost always required in patients needing treatment. Propranolol, the first-line medication for problematic IH, has no significant effect on CHs [7]. Moreover, the mechanistic investigation and drug screening of CHs are severely hampered by the absence of a vivo model and stable cell lines. For drug testing and pathogenesis research, many researchers still rely on classic two-dimensional in vitro cell cultures or animal models [8]. These models lack the intricate micro-environment of human tumors, such as cell‒cell and cell-extracellular matrix interactions, both of which are major determinants of cell fate and contribute to the failure of clinical medication translation [9, 10]. Organoids have developed into a viable research platform for drug development with the rapid advancement of regenerative medicine. Therefore, organoids have the ability to overcome the limitations of conventional models [8, 10]. Organoids are complex three-dimensional structures formed by a self-organizing process of stem cells or organ-specific progenitors [11]. Because organoids are composed of several cell types and comprise multicellular organ structures whose form and function resemble those of in vivo organs, organoids are regarded as a close replica of the human internal environment [12]. In recent years, numerous organoid models have been developed that have proven to be useful for high-throughput drug screening and mechanistic investigation [13, 14]. In our early investigation, we successfully developed a three-dimensional micro-tumor model of IH for mechanistic research and medication screening [15]. Based on our preliminary study [16], the organoid strategy manifested high potential to further depict mysterious NICH mechanisms.

One of the major challenges for building an NICH model is that it is difficult to obtain clinical samples. Until now, no investigations have claimed to successfully construct NICH cell lines or patient-derived xenograft (PDX) models. In the case of no existing in vivo or in vitro model for further discovering NICH genetic characteristics, the establishment of a specific research model for NICH is crucial for profoundly exploring etiological factors at the molecular level and cellular level tissue level. In recent years, the organoid method has been reported as a promising strategy to bring fresh air between clinical and basic research, including tumor and normal tissue. In a 3D culture environment, tissue-specific growth factors confer the capability to generate tissue-like organoids with specific features. To investigate the pathology of NICHs and prospective targeted medicines, reliable experimental models are essential. In this study, we successfully constructed patient-derived organoids (PDOs) from NICH and further evaluated the effectiveness of propranolol, sirolimus and trametinib in the treatment of NICH PDOs.

## Methods

This study was approved by the Ethics Committee of the West China Hospital of Sichuan University. Informed consent was obtained for experimentation with human subjects from all patients’ parents. All three tissues were obtained surgically at our hospital (Table S1), and the diagnosis of NICH was confirmed by pathological examinations and clinical characteristics.

### Human specimens

NICH and para-NICH tissues were obtained from patients who underwent surgery at the West China Hospital of Sichuan University. Clinical data are summarized in table S1 and figure S1. After excision, samples were temporarily stored in PBS containing antibiotics on ice. The samples were then transferred to a state-run Biotherapy lab, where they were cut into pieces smaller than 1 mm and cryopreserved at -80 °C Celsius.

### Establishment of organoids and passage

**Fig. 1:**
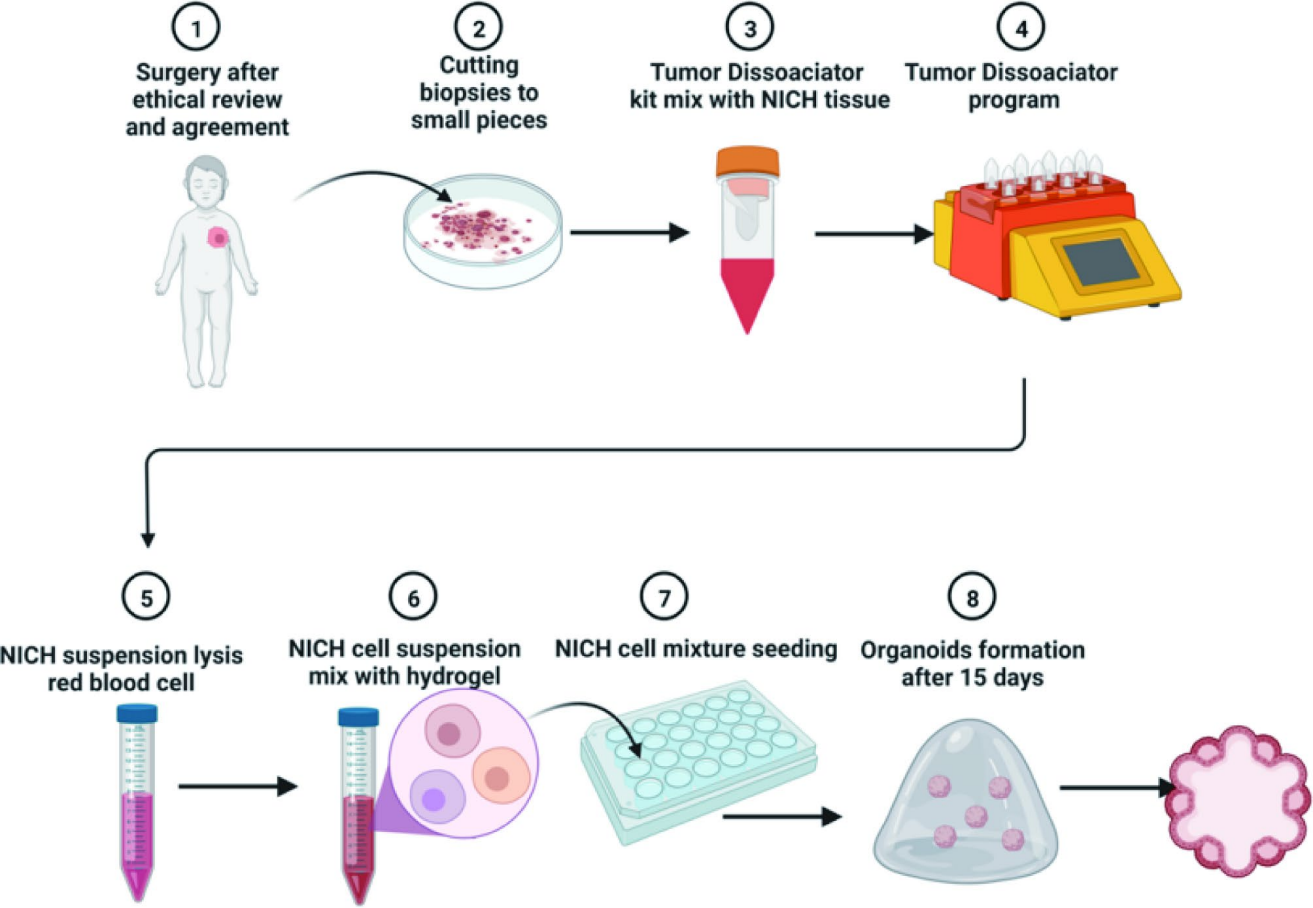
The process for constructing NICH organoids from NICH. Biopsies were harvested from patients, and organoids were constructed in eight steps

Patient-obtained tissue was pulverized and incubated in digestion solution (Miltenyi Tumor Dissociation Kit) (Fig. 1) for 60 min in accordance with Scheme A of the RWD Single-Cell Suspension Separator Manual (DSC-400). The suspension was filtered through a cell strainer (70 μm) and centrifuged at 600 × g for 5 min after digestion with Dulbecco’s modified Eagle medium (DMEM) containing 10% fetal bovine serum (FBS). The pellet was washed with cold Advanced DMEM/F12 (GIBCO, USA), and Red Blood Cell Lysis Buffer (20,220,811) was mixed with the cell pellet for 10 min, after which the pellet was centrifuged. After discarding the supernatant, Matrigel was used to resuspend the pellet (Corning, USA). Cells were seeded and cultured in a six-well suspension plate after counting (RWD, eC100) (10,000–20,000 cells per well). The organoid culture medium for NICH organoids consisted of Advanced DMEM/F12 medium, 1:50 B27 supplement, 1:100 N2 supplement, 1.25 mM N-acetyl-l-cysteine, 250 ng/ml Rspo-1, 1 mg/ml primocin, 100 ng/ml Noggin, 50 ng/ml EGF, 100 ng/ml FGF-10, 25 ng/ml recombinant human HGF, 10 mM Y27632, 10 mM nicotinamide, 5µM A83-01,10 µM forskolin, 10 ng/ml VEGF-a, Glutamax and 1% hydroxyethyl piperazine ethanesulfonic acid (HEPES).

The medium was changed every 4 days. The organoid numbers were counted on days 7, 10 and 13. Four views were randomly taken, and the organoid number was counted.

Matrigel was digested by TyplExpress (Gibco USA) and centrifuged at 300 g/min to collect the pellet. Then, the cells were resuspended at a ratio of 1:1.5 to 1:2 for three passages. Third passage organoids were divided into three groups: (1) Directly fixed organoids with Matrigel for further histology and IHC staining. (2) Matrigel was removed by TyplExpress, and PDO pellets were frozen at -80 °C for further gene analysis. (3) After removing the Matrigel, the pellets were resuspended in cell stock solution and stored at -80 °C in Celsius or liquid nitrogen.

### Histology and staining

Tissues and organoids were fixed with 4% neutral buffered formalin (Sigma–Aldrich) at room temperature for 48 h or 0.15 h separately. Paraffin embedding was performed as follows: samples went through a grade ethanol series, xylene and then paraffin. The embedded samples were cut into 5 μm sections and prepared for hematoxylin and eosin (H&E) and immunohistological (IHC) staining according to a standard protocol. For IHC, primary antibodies against CD31 (ab28364, 1:200), Factor VIII (ab275376, 1:200), GLUT-1 (ab150299, 1:200), VEGF-A (ab213244, 1:200), Vimentin (ab92547, 1:100) and VE-Cadherin (ab33168, 1:100) were used. Organoids were observed, and images were photographed with an Olympus BX51TRF microscope at different magnifications (HE 10X, IHC 40X).

### RNA sequencing analysis

Total RNA was extracted using the mirVana miRNA Isolation Kit (Ambion) following the manufacturer’s protocol. RNA integrity was evaluated using the Agilent 2100 Bioanalyzer (Agilent Technologies, Santa Clara, CA, USA). The samples with an RNA integrity number (RIN) ≥ 7 were subjected to subsequent analysis. The libraries were constructed using the TruSeq Stranded mRNA LT Sample Prep Kit (Illumina, San Diego, CA, USA) according to the manufacturer’s instructions. Then, these libraries were sequenced on the Illumina sequencing platform (HiSeqTM 2500 or Illumina HiSeq X Ten), and 125 bp/150 bp paired-end reads were generated. Then, NICH functional analysis of organoids was contrasted with multi-GSEA to calculate the enrichment score by applying GSEA 4.10 v (https://www.gsea-msigdb.org/gsea/index.jsp).

Differential expression analysis was carried out using the classic mode of the Bioconductor edgeR package with Bayes moderated dispersion parameter estimation [17]. Genes were considered differentially expressed (DEGs) when the absolute value of log2-fold-change was ≥ 1 and the false discovery rate (FDR) adjusted p value was < 0.05. Functional enrichment analysis, including Gene Ontology (GO) enrichment and Kyoto Encyclopedia of Genes and Genomes (KEGG) pathway analysis, was carried out by the Bioconductor cluster Profiler package based on the DEGs obtained in a comparison of different groups [18]. Based on the threshold p value < 0.05 and q-value < 0.05, GO terms and KEGG pathways with significant enrichment were screened out. Visualizations were created using ggplot, Goplot and path-view packages.

### Cell identification and drug validation

NICH organoids were seeded on 96-well plates at 200 organoid/well for further observation and verification of three clinical drugs. For derivation cell identification, organoids were seeded on 96-well plates, and photos were taken on days 5 and 10. Organoids were fixed on day 10 with PFA. Then, the cells were incubated in 0.2% Triton (20 µL in 10 ml PBS) for 5 min at room temperature and washed 1x for 5 min in PBS. Incubate cells with 0.1% sodium borohydride (10 mg in 10 ml of PBS) for 5–10 min at RT. The cells were washed 3 × 5–10 min in PBS. The cells were incubated with primary antibodies (in blocking solution) for 1 h at RT and then washed 3 × 5–10 min with PBS. The cells were incubated with secondary antibodies for 45 min − 1 h at RT (in the dark) and then washed 3 × 5–10 min with PBS. Add 90 µL DAPI (already diluted stock of 1:5000 in water in fridge) to each well and incubate for 5 min in the dark. The cells were washed 3x in PBS, and photos were taken by microscopy (Olympus BX51TRF).

NICH organoids were seeded on 96-well plates for 72 h to prepare for drug validation. Drugs were weighed and diluted in DMSO to final concentrations of 10 µg/L, 40 µg/L, 160 µg/L and control. Pictures were taken at 0, 24 and 48 h.

## Result

**Fig. 2:**
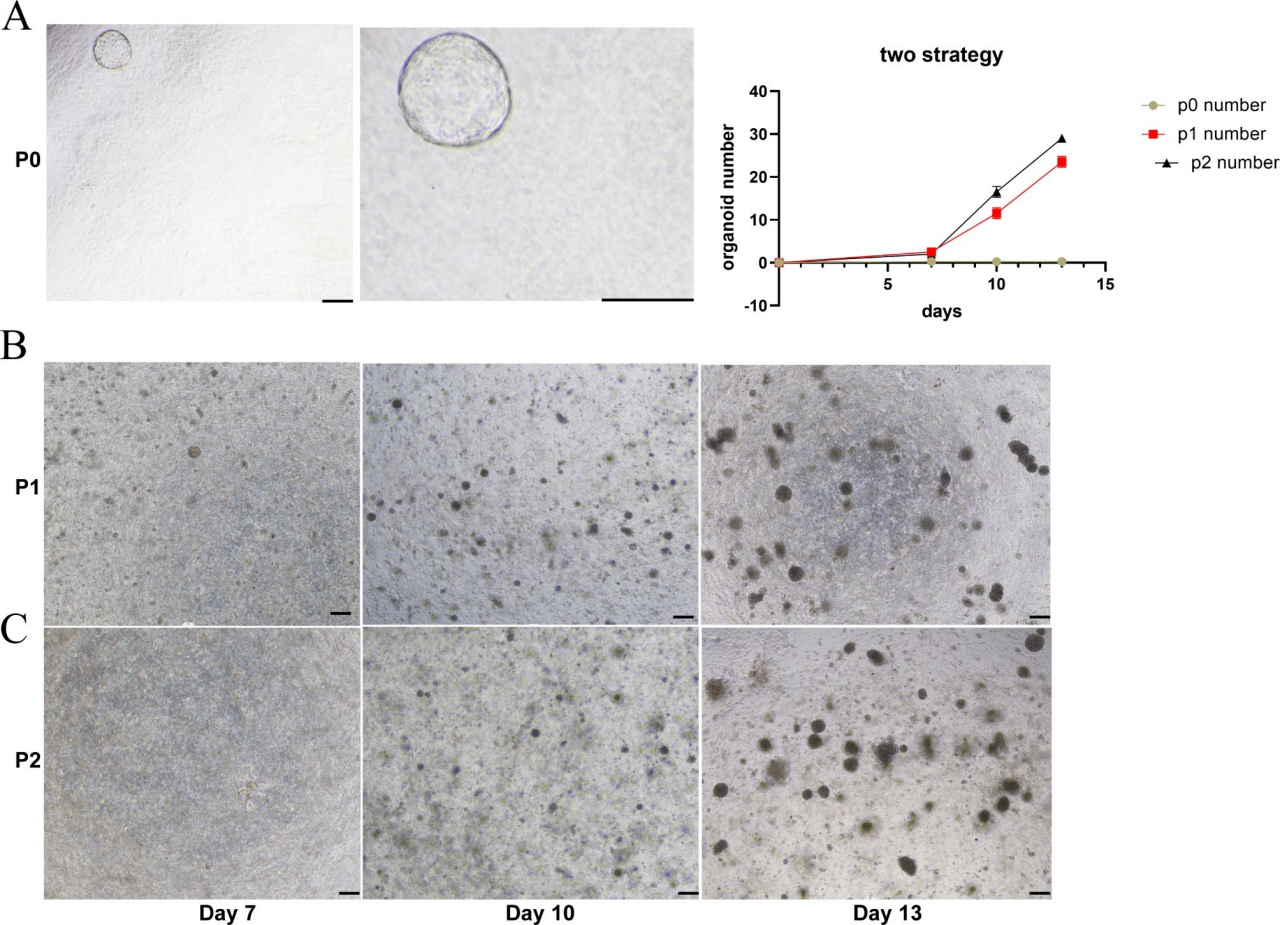
Two strategies for isolating cells. (**A**) NICH biospecimen incubated with diverse collagenases, one organoid growth. (**B**) and (**C**) Miltenyi cell separation kit with different single-cell systems. Scale bar = 100 μm

Generally, we focus on strategies from sample collection to organoid construction. According to the current situation in which no research has reported cell line construction, various digestion systems applied in tissue handling and adjustment in NICH organoids are essential for the efficiency and success rate. To construct the NICH organoid, we derived cells from tissue with diverse cell-derived systems. Initially, various collagenase subtypes and trypsinase with different periods were applied to separate NICH single cells. Then, the growth medium system of NICH organoids was based on multiple tissue and tumor organoid induction methods [19, 20]. Primarily, several collagenases reported in other organoid construction methods [21–23] were applied in NICH cell derivation (I, II, VI XI). However, only a single organoid was finally harvested (collagenase XI) (Fig. 2A).

Due to the very limited NICH organoid number in the first trial, new cell derivation methods were imperatively required to boost the cell derivation rate. Then, NICH cell number was significantly enhanced by a human Millet Cell Separation Kit (130-095-929, USA) with NICH organoid medium (Fig. 2B, C).

### Organoids faithfully recapitulate NICH at the morphological and histological levels

**Fig. 3:**
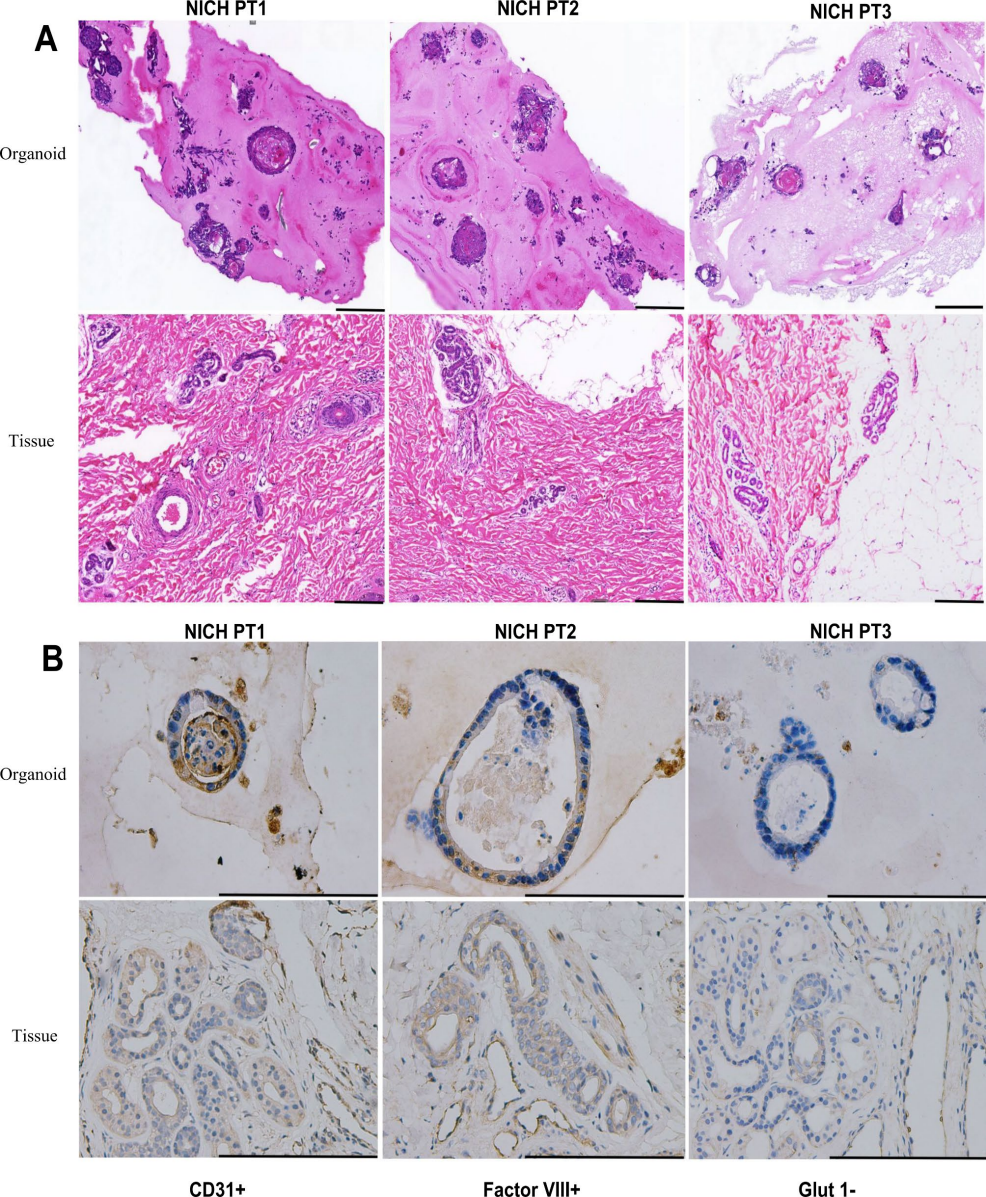
Features of NICH organoids by immumohistochemical staining (**A**) three NICH tissues and corresponding organoid HE staining. (**B**) Immunohistochemistry of CD31, factor VIII, and GLUT-1 in organoids and tissue. Scale bar = 100 μm

To compare NICH organoid tissue characteristics to their related tissues, we performed H&E staining (Fig. 3A) (organoids were fixed with Matrigel to ensure that the organoid and Matrigel showed antibody enrichment conditions reflecting the real growth microenvironment) and evaluated the expression of the main protein biomarkers for NICH (Fig. 3B), including PECAM-1, factor VIII, and GLUT-1. Of note, NICH organoids consist of the transformed endothelial cells of a tumor but do not include immune, vessel or connective tissue ingredients. Histological depiction of the primary NICH tissue used for organoid derivation uncovered major morphology if the organoid was captured or not from tissue. To boost the staining success rate, a new fixation strategy was applied to analyze NICH organoids with BME fixation [24, 25] to reduce organoid loss and maintain the micro-environment when organoids grew and developed. Specifically, BME displayed advantages in revealing the real environment, including key protein accumulation. The results of H&E showed high similarities in structure and cell staining between tissue and its derived organoid. Congenital hemangioma is positive for CD31 and CD34. Endothelial cells in CH, in contrast to those in IH, are usually negative for GLUT1 antibody [26]. The major discrepancy between NICH and IH is the biomarker GLUT-1, in which IH manifested as positive while NICH was negative or weakly positive. According to the results, NICH PDOs consistently illustrated tissue characteristics with GLUT-1 negativity, while both CD31 and Factor VIII were positive. This is a crucial histological demonstration in which we faithfully constructed a new model to uncover this mysterious hemangioma without any cell line or animal model.

### Transcriptomic characterization of NICH organoids

**Fig. 4:**
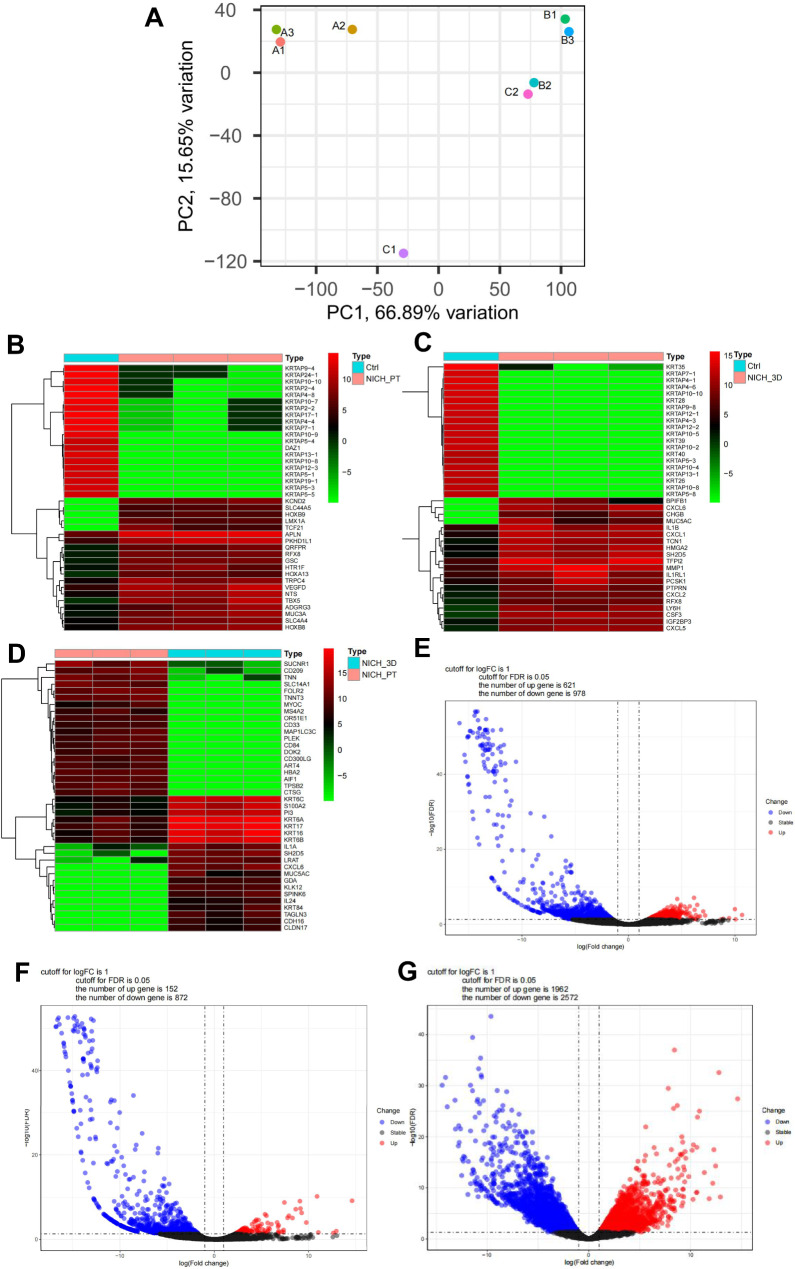
Transcriptome results of NICH organoids, NICH tissue and para-NICH tissue. (**A**) PCA of NICH PDOs (A1, A2, A3), NICH tissue (B1, B2, B3), and para-NICH tissue (C1, C2). Heatmap analysis of (**B**) NICH tissue with para-NICH tissue. (**C**) NICH PODs with para-NICH tissue. (**D**) NICH tissue with NICH PDOs. Volcano map of differentially expressed genes (log2FC > 1 and P-adjusted < 0.05 as a significant difference). Red dots indicate significantly upregulated genes, blue dots indicate significantly downregulated genes, and gray dots indicate nonsignificant genes. (**E**) NICH tissue compared with para-NICH tissue. (**F**) NICH PDOs compared with para-NICH tissue. (**G**) NICH organoid compared with NICH tissue

Three biopsies were collected and employed with the NICH organoid medium system for further gene expression data analysis. To assess organoid gene expression profiles, we performed RNA sequencing (RNA-seq) on 3 NICH-derived organoids (named group A), correlative NICH tissue (named group B) and para-NICH tissue (named group C). The PCA manifested a high similarity among the samples in groups A or B (Fig. 4A). However, the sample of group C3 was not included due to quality control. The sample of C2 in the PCA graph was so close to B2, indicating that the sampling distance may be too close for these biopsies during surgery. Then, C2 was excluded.

PCA revealed that three NICH PDOs generated from distinct NICH tissues exhibited a high degree of similarity, which clearly suggested that the method for constructing NICH organoids is reliable and consistent. Then, we specifically focused on NICH PDOs, NICH tissue and para-NICH tissue comparisons (Fig. 4B, C, D). The analysis of heatmaps revealed that genes linked to keratinization (KRTAP4-3, KRTAP4-6, KRTAP7-1, KRTAP1-4 KRTAP5-9, KRTAP17-1, KRTAP10-2, KRTAP10-1, KRTAP5-4) were considerably down regulated in groups A and B in comparison to group C. The volcano plot depicts gene up- and down-regulation trend pairwise comparisons in the three groups. 621 genes up 978 down in (group An in contrast with C(Fig. 4E) and 152 genes up and 872 genes down group B compared with C (Fig. 4F). However, when compared to group B, group A exhibited variation expression diversities (1962 up and 2572 down)(Fig. 4G) .This may be due to the reality that organoids still lack of skin tissue, blood and lymph vessels, in vivo real ECM environment, immnue system, all of which may lead to discrepancy in transcriptomes result.

### Gene functional analysis of NICH organoids

KEGG signaling pathway enrichment analysis indicated gene enrichment and correlation connections. In comparison to para-NICH tissue, the KEGG signaling pathway enrichment analysis indicated that chronic myeloid leukemia, vascular smooth muscle contraction, ECM receptor, focal adhesion, colorectal cancer, and small lung cancer signaling pathways were enriched in NICH tissue. KEGG function results between NICH PDOs and NICH tumors showed only slight enrichment in p53 and RNA polymerase in the organoid group, while NICH tumor focal adhesion and vascular smooth muscle contraction were in accordance with NICH characteristics (Figure S2 A B).

Specifically, related functional signal pathway KEGG enrichment was compared in the three groups. Tumor-related signaling pathways, including PI3K/AKT, MAPK, and RAS, were significantly enriched in both NICH tissues and NICH organoids (Figure S2 C, D).

### Further characteristics and drug validation of NICH organoids

When NICH organoids were seeded in 96-well plates, we surprisingly found that many cells split from the surrounding organoids (Fig. 6A). On day 10, the development was intrinsically tissue-like, with numerous organoid clones encircled by a complex microenvironment. This characteristic reveals that this rare vascular tumor may be very dynamic during its formative period.

**Fig. 5:**
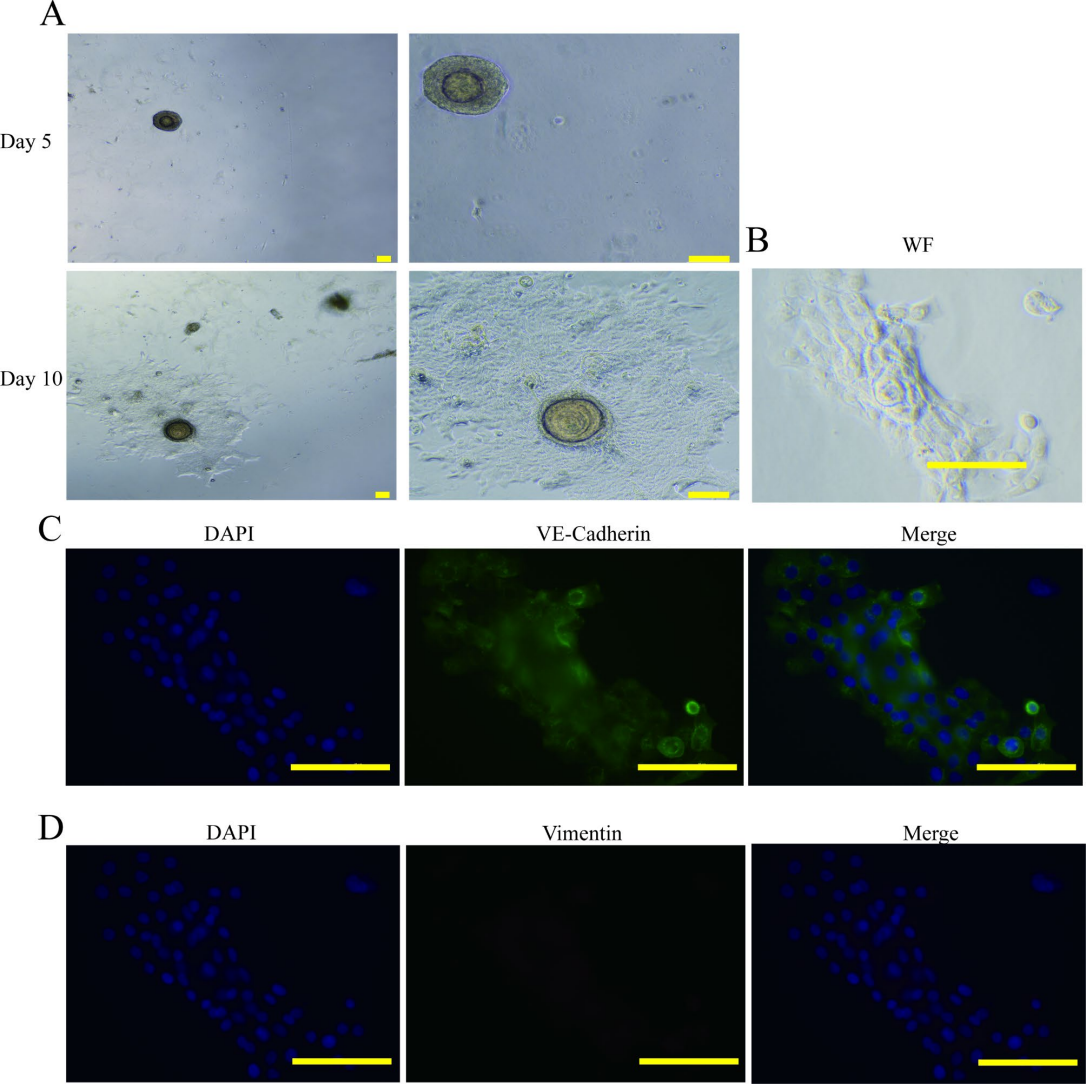
(**A**) NICH organoid seed in plates and ten days of cells derived from its organoid. (**B**) Bright field image, (**C**) VE-Cadherin in green and DAPI in blue, (**D**) Vimentin in red and DAPI in blue. Scale bar = 100 μm

To identify these cells, components of this mysterious microenvironment around organoids, immunocytochemistry was performed with two key biomarkers, VE-Cadherin in green and Vimentin in red. These antibodies effectively differentiate cell types, whether endothelial cells or fibroblasts, and there is a pathway for ECs to shift to fibrosis by endothelial-to-mesenchymal transition [27]. The results demonstrate that the cells derived from NICH organoids were positive for VE-Cadherin (Fig. 5C) but negative for vimentin (Fig. 5D), indicating that the main cells may be EC cells.

**Fig. 6:**
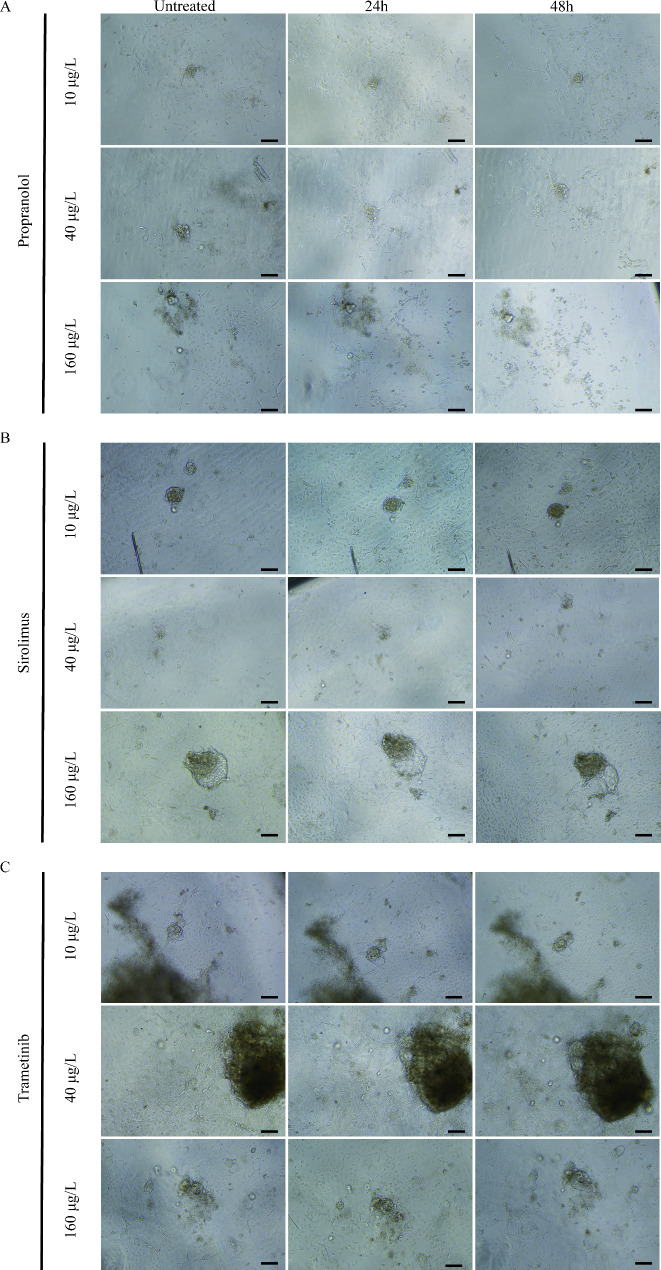
Clinical medical validation. (**A**), propranolol (**B**), sirolimus (**C**) propranolol. NICH organoids were treated with propranolol, sirolimus or trametinib for 24 and 48 h at 10 µg/ml, 40 µg/ml and 160 µg/ml. Scale bar = 100 μm

Propranolol, a nonselective beta-adrenergic receptor blocker, possibly accelerates the regression of proliferating IHs. However, the effect of propranolol is controversial in NICH therapy. Some research has claimed that propranolol is effective in the early stage of NICH, while other investigators have claimed that propranolol is invalid for NICH [28] Interestingly, in this study, the results indicated that propranolol (Fig. 6A) did not have a significant therapeutic effect on NICH organoids even at high dosages (160 µg/L). Moreover, neither sirolimus (Fig. 6B) nor trametinib (Fig. 6C) at different dosages had any appreciable effect on NICH organoid models, which indicates that more organoids need further drug screening.

## Discussion

The pathogenesis of NICH is still a mystery. Currently, due to the lack of an available cure strategy, as well as cell line and animal models to further unravel its mechanism and drug screening, the only therapy for NICH is surgery. Here, we report a novel method for NICH in vitro model instant construction. Initially, only a single NICH organoid was harvested from sample digestion and organoid build-up, but we are confident that the organoid strategy brings fresh air for this rare vascular tumor research. After optimization of the cell-derived method and growth system, the NICH organoid number was markedly enhanced, and the organoids faithfully captured NICH histological characteristics in HE (ICC). We found that GLUT-1 was negative, while CD31 and Factor VIII were positive in both organoids and tissues. Meanwhile, the biobank of NICH has started to be built to further uncover the molecular and cellular characteristics of NICH.

Previously, the low proliferation activity of NICH and the difficulty of cultivating primary tumor cells in vitro significantly impeded the study of its pathogenesis and drug screening. Organoid models have gained attention and have developed quickly over the past ten years as a result of the advancement of in vitro 3D cell culture technology, a better understanding of the extracellular matrix, and research into the stem cell niche [29]. Presently, many organoid models of normal tissues, such as the small intestine, lung, liver, and brain, have been established successfully and are widely used in mechanistic research and disease models [11]. Additionally, a number of tumor organoids, including colorectal cancer, non-small cell lung cancer, liver cancer, breast adenocarcinoma, and pancreatic cancer, have also been successfully established [30, 31]. The emergence of tumor organoids has brought revolutionary progress to in vitro tumor models and solved the difficult problems of primary tumor cells in culture and differentiation. Tumor organoids also provide new ideas for tumor research, particularly in the highly promising field of personalized tumor treatment. In the present study, NICH organoids were successfully established after the cell-derived method and growth system were successfully optimized as described in this study. As a 3D culture model in vitro, organoids mimic the structure and function of in vivo tumor tissues by exhibiting cell‒cell and cell‒ECM interactions that share similar pathophysiological characteristics [30]. In this study, the HE results revealed that the structure and cell staining of the tumor tissue and the derived organoids were remarkably similar. Moreover, the results showed that CD31, Factor VIII and C-myc all displayed positive tissue characteristics, while GLUT-1 was consistently demonstrated to be negative. The results of the PCA showed that the three organoid lines from different patients shared high similarities, indicating NICH organoid stability.

In most cases, NICH can be managed with supportive care and an expectant approach. However, medical treatments were not supported by evidence in cases requiring intervention, particularly for large NICHs that may be linked to internal malformations or result in functional impairment, such as plagiocephaly, positional torticollis and difficulty breathing [4, 32, 33]. Propranolol, vincristine and corticosteroids have been utilized with limited effectiveness [32]. Embolization is usually utilized as an additional bleeding control method [34, 35]. Surgical excision is the only treatment method that has been demonstrated to be definitive [36, 37]. There is a critical knowledge gap in that patients diagnosed with NICH do not have access to adequate medical therapy alternatives [32]. Additional research is required to identify optimal medical therapies to enhance other interventional approaches for high-risk patients [32]. Propranolol has been demonstrated to be successful in the treatment of IH, and it has been claimed that NICH can be treated with propranolol alone without causing major side effects [28]. However, additional studies revealed that propranolol was ineffective for treating NICH or PICH 32, 34–36]. In this investigation, we discovered no substantial inhibitory effect of propranolol on NICH organoids. Oncogenes that stimulate the MAPK/MEK/Ras pathway, GNAQ and GNA11, have been related to CH [1, 32, 38].

## Conclusion

According to preliminary research, uveal melanoma with mutations in GNAQ and GNA11 is responsive to MEK inhibitors and could be used as a model for medical treatment for CH [32, 39]. The mTOR inhibitor sirolimus may also be worth studying in view of the established interaction and coregulation of PIK3CA/AKT/mTOR with the MAPK/MEK/Ras pathway, as well as its successful usage in treating complex vascular malformations [32, 40, 41]. In our previous study, sirolimus was found to be efficacious in treating kaposiform hemangioendothelioma [42, 43]. The significantly enriched MAPK signaling pathway was also identified in our sequencing results for NICH. Regrettably, NICH-derived organoids were not inhibited by sirolimus and trametinib.

Further efforts are needed to better understand the mechanism of NICH. The development of novel therapeutics and innovative approaches for NICH is required to enhance the response and reduce the risk of long-term sequelae.

## Study limitations

Based on this unique NICH PDO model, we passaged the organoids three times and then checked the histology and transcriptomes. Small sample size, selection bias, and deviation in sampling limited this research.

More passages will be performed to ensure culture system stability and uncover the mechanism during NICH organoid development. Additionally, drug screening will be conducted to identify appropriate candidates for patients.

## Electronic supplementary material

Below is the link to the electronic supplementary material.

**Additional file 1: Figure S1:** Sample collection from three patients. Three patients? NICH tissue sizes and positions.

**Additional file 2: Figure S2:** Transcriptome result functional comparison. (A) Para-NICH tissue compared with NICH tissue, (B) NICH organoid compared with NICH tissue. (C) Bubble graph of KEGG signaling pathway enrichment between NICH tissue and para-NICH tissue. (D) Bubble graph of KEGG signaling pathway enrichment between NICH organoids and NICH tissue. (E) 20 signal pathway enrichment in NICH organoids. (F) 20 signal pathway enrichment in PI3K/AKT in NICH.

**Additional file 3: Table S1**: Clinical features of congenital hemangioma

## Data Availability

All relevant data will be freely available post-publication to any scientist that show interest or made a request.
