# Supplementary figures and images for: Novel organoid construction strategy for non-involuting congenital hemangioma for drug validation
Source: J Biol Eng. 2023 Apr 27;17:32. doi: 10.1186/s13036-023-00348-6 (PMC10142414; doi:10.1186/s13036-023-00348-6)

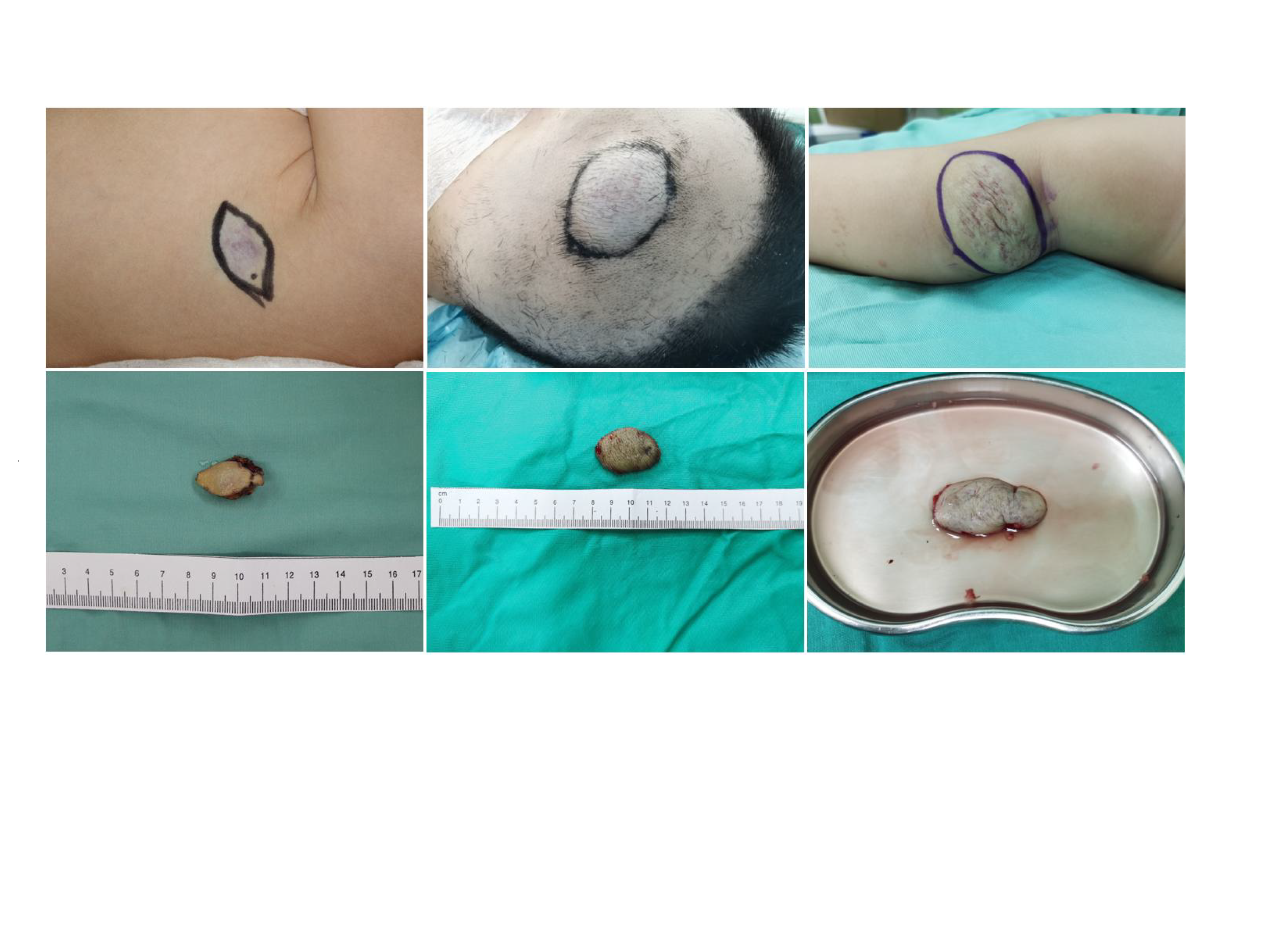

Supplement: Supplementary file 1 — Additional file 1: Figure S1: Sample collection from three patients. Three patients? NICH tissue sizes and positions. [file 13036_2023_348_MOESM1_ESM.tif]
