# Supplementary material for: Novel organoid construction strategy for non-involuting congenital hemangioma for drug validation
Source: J Biol Eng. 2023 Apr 27;17:32. doi: 10.1186/s13036-023-00348-6 (PMC10142414; doi:10.1186/s13036-023-00348-6)
Supplement: Supplementary file 3 — Additional file 3: Table S1: Clinical features of congenital hemangioma [file 13036_2023_348_MOESM3_ESM.doc]

**Table S1 Clinical features of congenital hemangioma**

|  | Age (year) | Location | Size (cm) | Sex |
| --- | --- | --- | --- | --- |
| Patient 1 | 4 | Armpit | 3 | Male |
| Patient 2 | 4 | Occipitalia | 4 | Male |
| Patient 3 | 1 | Lower leg | 7 | Female |
